# The Impact of Sex Hormones on Transcranial Magnetic Stimulation Against the Oxidative Stress in the Pathogenesis of Multiple Sclerosis

**DOI:** 10.3390/biom15121714

**Published:** 2025-12-10

**Authors:** Begoña M. Escribano, Manuel E. Valdevira, Ana Muñoz-Jurado, Montse Feijóo, Eduardo Agüera, Javier Caballero-Villarraso, Manuel LaTorre, Ana I. Giraldo, Abel Santamaría, Isaac Túnez

**Affiliations:** 1 Department of Cell Biology, Physiology and Immunology, Faculty of Veterinary Medicine, University of Cordoba, 14071 Cordoba, Spain; 2 Maimonides Institute for Research in Biomedicine of Cordoba (IMIBIC), 14004 Cordoba, Spain; 3 Cooperative Research Thematic Excellent Network on Brain Stimulation (REDESTIM), Ministery of Science, Innovation and Universities, 28046 Madrid, Spain; 4 Department of Biochemistry and Molecular Biology, Faculty of Medicine and Nursing, University of Cordoba, 14004 Cordoba, Spain; 5 Department of Medical and Surgery Sciences, Faculty of Medicine and Nursing, University of Cordoba, 14004 Cordoba, Spain; 6 Neurology Service, Reina Sofia University Hospital, 14004 Cordoba, Spain; 7 Analysis Service, Reina Sofia University Hospital, 14004 Cordoba, Spain; 8 Department of Animal Production, Faculty of Veterinary Medicine, University of Cordoba, 14071 Cordoba, Spain; 9 Faculty of Sciences, National Autonomous University of Mexico, Mexico City 04510, Mexico

**Keywords:** estrogens, experimental autoimmune encephalomyelitis, hormone replacement therapies, ovariectomy, progesterone

## Abstract

Background: Multiple sclerosis (MS) is an autoimmune neurodegenerative disease with a higher prevalence in women. While puberty appears to act as a trigger for MS, menopause has no clear effects on disease progression. Many studies have shown that transcranial magnetic stimulation (TMS) is a potential antioxidant treatment for MS, but the sexual hormones have been identified as a potential factor affecting TMS response by affecting cortical excitability and possibly clinical outcomes. Methods: The aim of this study was to test the effect of estrogen, progesterone, and testosterone hormonal supplementation as adjuvants to TMS treatment of experimental autoimmune encephalomyelitis (EAE), an experimental model of MS. The effects of the three hormones were also tested as replacement therapy in ovariectomized rats treated with TMS. Clinical signs of the disease, as well as disease-induced oxidative stress and antioxidant defenses of the glutathione system, were evaluated. Results: TMS alone, without supplements or replacement therapies, is effective against oxidative stress caused by EAE. Estrogen and progesterone replacement therapy is useful to enhance the role of TMS in ovariectomized rats, activating antioxidant defenses and improving clinical signs of the disease. Conclusions: TMS is effective in the treatment of MS, but its role could be enhanced, using hormone replacement therapy with estrogens and/or progesterone.

## 1. Introductions

Transcranial magnetic stimulation (TMS) has experienced unstoppable growth as a therapeutic strategy for neuropsychiatric disorders, such as major depressive disorder and obsessive-compulsive disorder, over the past 20 years. Various TMS pulse protocols and several device companies have received approval for therapeutic use in populations across multiple continents [1]. Furthermore, TMS has shown promise as a potential treatment for multiple sclerosis (MS) [2], as it improves nerve conduction function and produces anti-inflammatory and antioxidant effects [3].

Multiple sclerosis (MS) is a chronic autoimmune, inflammatory, and neurodegenerative disease that affects the central nervous system (CNS). MS affects the myelin sheath of CNS neurons, leading to axon injury, neuronal death, and neurological progression [4]. The mechanisms involved in the pathogenesis of MS have not been fully elucidated [5]. Currently, it is known that one of the main triggers of MS is the exacerbated activation of the immune system, where lymphocytes attack the myelin [6]. These immune responses lead to inflammatory reactions, oxidative stress, neuronal energy deficit, loss of myelin trophic support, etc. [3].

Another important factor in the pathogenesis of MS is oxidative stress [3], and it can be inferred that, at least in part, the beneficial effect of TMS on MS is due to its antioxidant effect. TMS induces the translocation of Nrf2 nuclear factor, basic leucine zipper protein that may regulate the expression of antioxidant proteins, causing antioxidant gene expression, which recovers antioxidant balance and blocks neuroinflammation. This process may prompt an increase in cell density in the brain in probable relation to a reduced expression of the pro-apoptotic protein Bax and an increase in the anti-apoptotic protein Bcl-2 [7]. TMS has the ability to decrease lipid peroxidation products and protein carbonylation. It also increases the reduced glutathione/oxidized glutathione ratio [8]. TMS has demonstrated a more active role against oxidative stress and clinical damage in experimental autoimmune encephalomyelitis (EAE) and MS than other traditional treatments such as natalizumab, dimethyl fumarate, and dexamethasone [8,9], acting alone or with adjuvants such as S-allyl cysteine [10]. On the other hand, sexual dimorphism plays a key role in brain development and therefore presents greater complexity in behavioral outcomes, both under normal conditions and under disease conditions, such as neurodegenerative disorders [11]. In MS, the influence of biological sex on incidence, prevalence, course, severity, and prognosis has been described [12,13,14]. Although MS disproportionately affects more women than men [12,13,14,15], women more often present with a more benign course, with predominantly sensory symptoms, and fewer inflammatory relapses [12,14]. On the contrary, men present more motor symptoms, cognitive impairment, and overall a worse progression [14,16].

The role of sex hormones appears to be important in MS. An example of this is that MS is not very prevalent before puberty, but may begin to manifest after puberty, more frequently in girls, highlighting a possible role of sex hormones in MS [17]. This has also been demonstrated in mice with EAE [17], an experimental animal for MS. Post-pubertal female mice showed a higher incidence of both active and adoptive transfer EAE compared to age-matched pre-pubertal counterparts that had been oophorectomized prior to puberty. However, the incidence of EAE did not vary between age-matched prepubertal and post-pubertal males [17]. On the other hand, in women, studies to date have revealed contradictory results regarding menopause. Some studies report no difference in outcomes after the onset of menopause, and others report worsening MS symptoms [18], disease progression, and increased brain atrophy on radiological tests [19].

The role of exogenous hormone replacement therapy to improve menopause-related symptoms and promote neuroprotection in MS remains unclear [18,19]. This is because current recommendations limit the use of these medications beyond 60 years or 10 years of exposure [19]. However, sex hormones have been suggested to have immunomodulatory, neuroprotective, anti-inflammatory, and promyelinating effects in preclinical and clinical trials [18,20,21]. In addition, an ever-increasing clinical data demonstrates that sex hormones (estrogens and testosterone) are therapeutically effective in animal models of MS [22], such as EAE rats. An example of this is that during the peak of EAE, a prolonged state of diestrus has been recorded, with maintenance of the corpora lutea, high intraovarian levels of progesterone, and a decrease in ovarian levels of testosterone and estradiol. In contrast, serum testosterone levels were slightly increased [23].

However, sex hormones have been identified as a potential factor affecting TMS response, mediating cortical excitability and possibly clinical outcomes. Specifically, in a review article by Rivas-Grajales et al. 2023, higher levels of estrogen and testosterone were associated with greater cortical excitability, whereas higher levels of progesterone were associated with lower cortical excitability [24]. However, the role of sex hormones in the antioxidant function of TMS has not been reviewed.

The proposal of this study is novel as it is based on the role of sex hormones in the application of transcranial magnetic stimulation in intact EAE rats and others undergoing menopause by ovariectomy. On the one hand, we investigate the role of estrogens, progesterone, and testosterone as adjuvants of TMS. On the other hand, we want to evaluate the role of hormone replacement therapy of estrogen, progesterone, and testosterone in ovariectomized EAE rats, also subjected to treatment with TMS.

## 2. Materials and Methods

### 2.1. Animals

The animals were obtained from the Animal Experimentation (University of Cordoba, Cordoba, Spain). All experiments were approved by the Bioethics Committee at University of Cordoba (30/03/2017/053) and carried out according to the guidelines of the Directive 86/609/ECC approved by the European Communities Council, the Directive 2010/63/UE del European Parliament and Council and RD 53/2013 passed by Presidency Minister of Spain (BOE 8 February 2013).

Twelve-week-old adult Dark Agouti female rats weighing between 200 and 230 g were used. Each group was housed individually in plastic cages. They were maintained under controlled conditions of temperature (21 ± 2 °C) and illumination (12 h light/12 h dark cycle, lights on at 08:00 h), and were provided with standard AIN-93G rat daily diet chow [25] and water ad libitum.

### 2.2. EAE Induction

EAE induction was performed by injecting subcutaneously, at the dorsal base of the tail, 100 mL of a solution containing 150 mg of myelin oligodendrocyte glycoprotein (MOG) (fragment 35–55; Sigma–Aldrich, Madrid, Spain) in phosphate buffered saline (PBS) emulsified 1:1 in complete Freund’s adjuvant (Sigma-Aldrich, St. Louis, MO, USA) completed with 400 mg of heat-inactivated *Mycobacterium tuberculosis* (H37Ra, DIFCO, Detroit, MI, USA).

### 2.3. Experimental Groups and Treatments

Five rats were included in each experimental group. The groups were as follows: control; vehicle; EAE; EAE+Mock; EAE+TMS; EAE+TMS+P (EAE+TMS+progesterone); EAE+TMS+E (EAE+TMS+estrogens); EAE+TMS+T (EAE+TMS+testosterone); EAE+TMS+Sham; EAE+TMS+OVX (EAE+TMS+ ovariectomy); EAE+TMS+OVX+P; EAE+TMS+OVX+E and EAE+TMS+OVX+T.

All treatments were started at the same time, starting on day 14 after EAE induction in the corresponding groups [26,27,28]. All treatments lasted 21 days.

The control group received no treatment. The vehicle group was administered 100 mL of complete Freund’s adjuvant without MOG.

TMS treatment was carried out as follows: the animals were placed in cylindrical plastic cages designed to keep them immobile. Each coil consisted of 1000 turns of enameled copper wire (7 cm diameter) contained in plastic boxes (10.5 × 10.5 × 3.5 cm). A pair of Helmholtz coils generated the fields (Magnetotherapia S.A., Mexico). The two coils were placed dorsally and ventrally to the head. The distance between each coil and the midpoint of the head was approximately 6 cm. The stimulation consisted of an oscillatory magnetic field in the form of a sine wave with a frequency of 60 Hz and an amplitude of 0.7 mT (ELEMF) applied for two hours in the morning, once a day, five days a week (Monday to Friday), for 21 days, in order to simulate clinical practice [7,8,10,29,30]. The animals in the Mock group were treated in the same way as those in the TMS group, but without receiving real stimulation. The objective of having this group was to study the effects of immobilization stress caused by plastic cages [7,8,10,30].

Rats were ovariectomized (OVX), under anesthesia and asepsis, following the bilateral procedure described by Poumeau-Delille (1953), or sham-operated (Sham) [31]. After a 14-day recovery period [32], the rats were randomly assigned to their groups for EAE induction. This resulted in approximately 28 days between the ovariectomy and the application of treatments, a safety margin for the absence of an ovarian cycle, which can take up to 20 days [33,34].

The administered hormonal dose of estrogens (17-β estradiol) was 2.5 mg/kg weight subcutaneous (s.c) [33], and progesterone was 8 mg/kg weight s.c. [34] and testosterone was 0.5 mg/kg weight s.c. [35]. The rats were injected for 5 days a week over 21 days, starting on day 14, coinciding with the application of TMS, to verify the role of hormone therapy on the antioxidant capacity of TMS. All reagents were purchased from Sigma (St. Louis, MO, USA). The doses used for estrogen and testosterone have proven effective against oxidative stress and brain cell death in ovariectomized rats with Huntington’s disease [33,35]. Progesterone, at the same dose, has also been shown to be beneficial for ovarian health when used as an adjuvant against ovarian damage in patients undergoing cancer chemotherapy with cisplatin [34].

### 2.4. Sample Preparation

The animals were sacrificed on day 35 after intraperitoneal anesthesia with 75 mg/kg of ketamine (Imalgene^®^ 100 mg/mL, Merial Laboratorios, Barcelona, Spain). Subsequently, they were decapitated. The brain and spinal cord were removed cold (0–4 °C) and weighed. They were then homogenized using a mechanical homogenizer (Tempest Virtis, Labsquip, Markham, ON, Canada) in tris buffer (20 mM) at pH 7.4.

### 2.5. Clinical Status

The animals were monitored at 14 and 35 days and scored in accordance with the following severity scale: 0: no signs, 1: tail paralysis, 2: weakness in hind legs, 3: paralysis in hind legs, 4: paralysis in hind legs and weakness in front legs, 5: quadriplegic [36]. The increase between the score at 35 days and the score at 14 days was established.

### 2.6. Parameters of Oxidative Stress

In the brain and spinal cord were determined: total glutathione (tG; nmol/mg protein), reduced glutathione (GSH; nmol/mg protein), oxidized glutathione (GSSG; nmol/mg protein), glutathione peroxidase (GPx; nmol/mg protein), and the ratio between GSH/GSSG. All parameters were analyzed by spectrophotometry with Bioxytech S.A. reagents (Oxis International; Portland, OR, USA) and with a Shimadzu spectrophotometer (UV 1603; Kyoto, Japan). The reagent kits of Bioxytech S.A. reagents (Oxis International, Portland, OR, USA) were GSH 420 (tG), GSH 400 (GSH), and GSH 412 (GSSG). To determine GPx (C.E.:1.11.1.12), the Flohé and Günzler method was used [37].

Lipid peroxidation products (LPO; nmol/mg protein) were determined with the LPO 586 kit (Bioxytech S.A. reagents, Oxis International, Portland, OR, USA). The assay protocol used a chromogenic reagent that reacts with the LPPs malondialdehyde and 4-hydroxyalkenals at 45 ± 1 °C, yielding a stable chromophore with a maximum absorbance at 586 nm.

Carbonylated proteins (CP) (nmol/g protein) were measured as an indicator of protein oxidative stress. Carbonyl content was evaluated using the Levine et al. method in a Shimadzu spectrophotometer (UV-1603; Kyoto, Japan) at a wavelength of 360 nm [38].

### 2.7. Statistical Analysis

Data were expressed as mean ± Standard deviation (SD). All groups showed a normal distribution, so a one-way ANOVA and Bonferroni post hoc were used to determine which specific groups had significant differences (SPSS INC., Version 25 for Windows). Comparisons were made between the EAE group with the control group and between the rest of the groups with EAE, EAE+TMS, and EAE+TMS+OVX. The level of significance was *p* < 0.05.

## 3. Results

In the evaluation of the results, the vehicle groups are not described as they do not differ from the control group. The results of the EAE+Mock group are not described either, as no differences were found with the EAE group, nor the results of EAE+TMS+Sham, as no differences were found with the EAE+TMS group (supplementary materials).

### 3.1. Clinical Status

**Figure 1:**
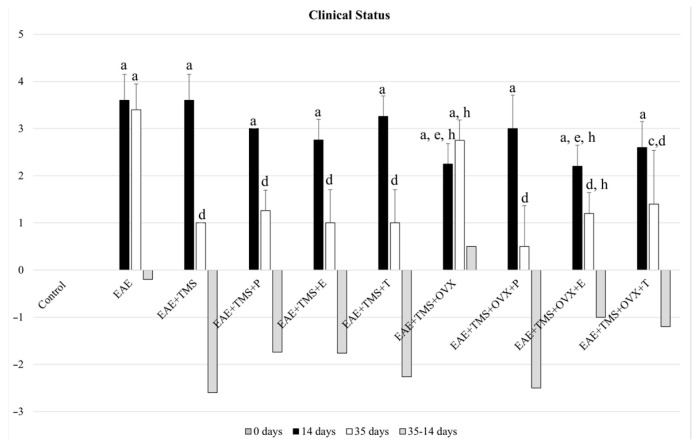
Mean ± standard deviation of the change in clinical score in rats at 14 days (without application of treatments), after EAE induction with MOG; at 35 days of illness after the application of the different treatments (from day 14 to day 35, except in the EAE group) and increase between the score at 35 days—and at 14 days, in the groups: control; EAE; EAE+TMS; EAE+TMS+P; EAE+TMS+E; EAE+TMS+T; EAE+TMS+OVX; EAE+TMS+OVX+P; EAE+TMS+OVX+E and EAE+TMS+OVX+T. The animals were monitored at 14 and 35 days and scored according to the following severity scale: 0: no signs, 1: tail paralysis, 2: hind leg weakness, 3: hind leg paralysis, 4: hind leg paralysis and front leg weakness, 5: quadriplegic. The control group has a score of 0. ^a^ *p* < 0.001 vs. control; ^c^ *p* < 0.05 vs. control; ^d^ *p* < 0.001 vs. EAE; ^e^ *p* < 0.01 vs. EAE; ^h^ *p* < 0.01 vs. EAE+TMS. EAE: experimental autoimmune encephalomyelitis; MOG: myelin oligodendrocyte glycoprotein; TMS: transcranial magnetic stimulation; OVX: ovariectomized rats; P: progesterone; E: estrogen; T: testosterone.

At 14 days after EAE induction, all groups show values above two on the Perez-Nievas et al. (2010) scale [36]. At 35 days, the values of the Pérez-Nievas et al. (2010) [36] scale were significantly reduced in all groups, with respect to the EAE group, except in the EAE+TMS+OVX. This group shows significant differences with the control group (*p* < 0.001) but not with the EAE group, although the clinical score in this group shows a tendency to decrease from 20% with respect to the EAE group at 35 days. EAE+ TMS+OVX and EAE+TMS+OVX+E also show worse clinical status than the EAE+TMS group, with higher scores on the Pérez-Nievas et al. (2010) [36] scale. EAE+TMS+OVX+T improves the EAE clinical score, but without recovering the control values (Figure 1).

### 3.2. Glutathione Redox System

**Figure 2:**
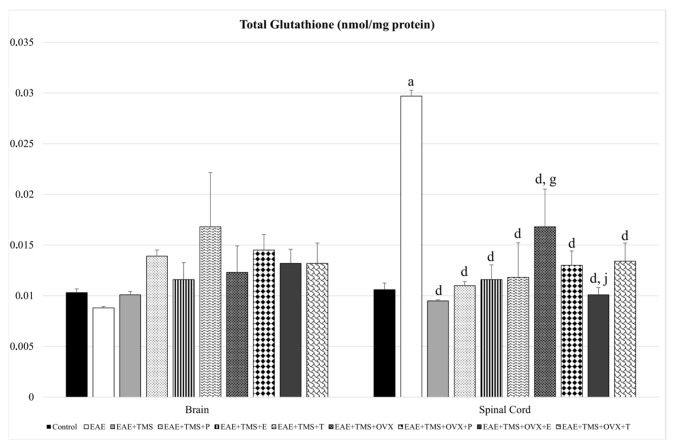
Mean ± standard deviation in total glutathione (nmol/mg protein) in EAE rats treated with TMS plus hormonal therapy in the following groups: control; EAE; EAE+TMS; EAE+TMS+P; EAE+TMS+E; EAE+TMS+T; EAE+TMS+OVX; EAE+TMS+OVX+P; EAE+TMS+OVX+E and EAE+TMS+OVX+T in brain and spinal cord. ^a^ *p* < 0.001 vs. control; ^d^ *p* < 0.001 vs. EAE; ^g^ *p* < 0.001 vs. EAE+TMS; ^j^ *p* < 0.001 vs. EAE+TMS+OVX. EAE: experimental autoimmune encephalomyelitis; TMS: transcranial magnetic stimulation; OVX: ovariectomized rats; P: progesterone; E: estrogen; T: testosterone.

In the spinal cord, EAE significantly increases (*p* < 0.001) its tG values compared to the control. With the different treatments (TMS, TMS+ovariectomy, and hormones), the levels of total glutathione are significantly reduced compared to EAE (*p* < 0.001), with the values of EAE+TMS+OVX being significantly higher (*p* < 0.001) than those of EAE+TMS. The results of EAE+TMS+OVX+E are lower (*p* < 0.001) than those of EAE+TMS+OVX. There are no significant changes in tG in the brain (Figure 2).

**Figure 3:**
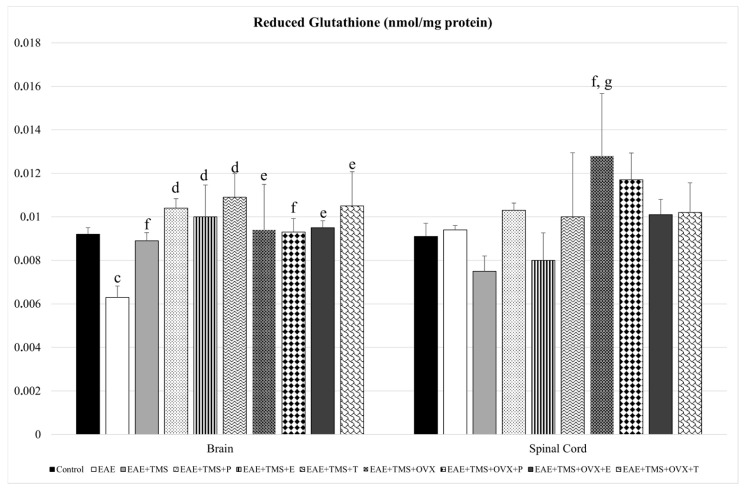
Mean ± standard deviation in reduced glutathione (nmol/mg protein) in EAE rats treated with TMS plus hormonal therapy in the following groups: control; EAE; EAE+TMS; EAE+TMS+P; EAE+TMS+E; EAE+TMS+T; EAE+TMS+OVX; EAE+TMS+OVX+P; EAE+TMS+OVX+E and EAE+TMS+OVX+T in brain and spinal cord. ^c^ *p* < 0.05 vs. control; ^d^ *p* < 0.001 vs. EAE; ^e^ *p* < 0.01 vs. EAE; ^f^ *p* < 0.05 vs. EAE; ^g^ *p* < 0.001 vs. EAE+TMS. EAE: experimental autoimmune encephalomyelitis; TMS: transcranial magnetic stimulation; OVX: ovariectomized rats; P: progesterone; E: estrogen; T: testosterone.

With respect to GSH, EAE+TMS+OVX rats show significantly higher values compared to EAE (*p* < 0.05) and EAE+TMS (*p* < 0.001) in the spinal cord. In the brain, EAE decreases its GSH values with respect to control (*p* < 0.05), while the use of all treatments (TMS, OVX, and hormones) increases GSH levels with respect to EAE (Figure 3).

**Figure 4:**
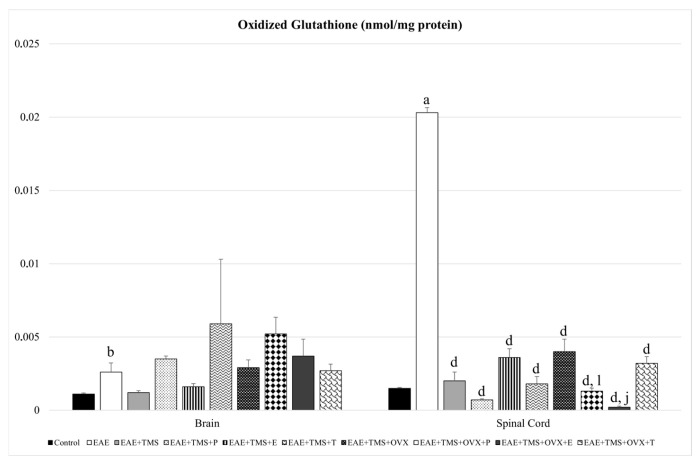
Mean ± standard deviation in oxidized glutathione (nmol/mg protein) in EAE rats treated with TMS plus hormonal therapy in the following groups: control; EAE; EAE+TMS; EAE+TMS+P; EAE+TMS+E; EAE+TMS+T; EAE+TMS+OVX; EAE+TMS+OVX+P; EAE+TMS+OVX+E and EAE+TMS+OVX+T in brain and spinal cord. ^a^ *p* < 0.001 vs. control; ^b^ *p* < 0.01 vs. control; ^d^ *p* < 0.001 vs. EAE; ^j^ *p* < 0.001 vs. EAE+TMS+OVX; ^l^ *p* < 0.05 vs. EAE+TMS+OVX. EAE: experimental autoimmune encephalomyelitis; TMS: transcranial magnetic stimulation; OVX: ovariectomized rats; P: progesterone; E: estrogen; T: testosterone.

GSSG increases in the brain (*p* < 0.01) and spinal cord (*p* < 0.001) for EAE compared to control. The use of different treatments (TMS, OVX, and hormones) in the spinal cord significantly reduces GSSG values with respect to EAE. Progesterone and estrogens in the spinal cord significantly reduce GSSG levels in rats treated with TMS and OVX (EAE+TMS+OVX+P and EAE+TMS+OVX+E) compared to EAE+TMS+OVX without hormonal treatment (Figure 4).

**Figure 5:**
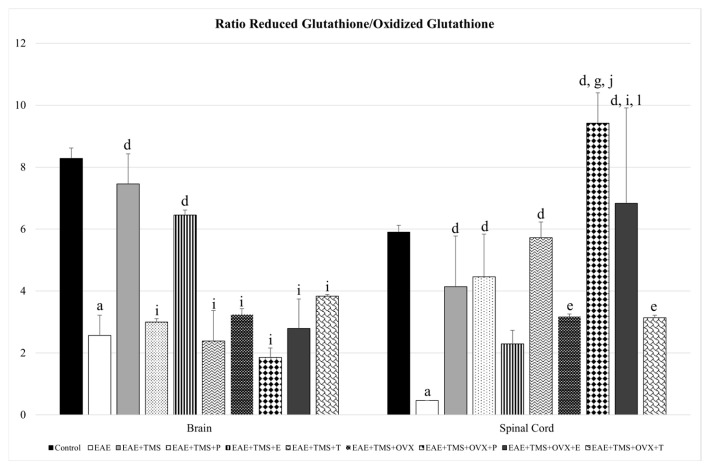
Mean ± standard deviation in ratio reduced glutathione/oxidized glutathione in EAE rats treated with TMS plus hormonal therapy in the following groups: control; EAE; EAE+TMS; EAE+TMS+P; EAE+TMS+E; EAE+TMS+T; EAE+TMS+OVX; EAE+TMS+OVX+P; EAE+TMS+OVX+E and EAE+TMS+OVX+T in brain and spinal cord. ^a^ *p* < 0.001 vs. control; ^d^ *p* < 0.001 vs. EAE; ^e^ *p* < 0.01 vs. EAE; ^g^ *p* < 0.001 vs. EAE+TMS; ^i^ *p* < 0.05 vs. EAE+TMS; ^j^ *p* < 0.001 vs. EAE+TMS+OVX; ^l^ *p* < 0.05 vs. EAE+TMS+OVX. EAE: experimental autoimmune encephalomyelitis; TMS: transcranial magnetic stimulation; OVX: ovariectomized rats; P: progesterone; E: estrogen; T: testosterone.

The GSH/GSSG ratio decreases significantly with EAE with respect to control (*p* < 0.001) in both the brain and the spinal cord. In the latter, all treatments (TMS, OVX, and hormones) significantly increase the GSH/GSSG ratio with respect to EAE, except the EAE+TMS+E group, which shows no significant changes. EAE+TMS+OVX+P and EAE+TMS+OVX+E rats show significantly higher ratios than EAE+TMS and EAE+TMS+OVX, both in the spinal cord. In the brain, only treatments with TMS (EAE+TMS) and TMS plus estrogens (EAE+TMS+E) significantly increase the ratio with respect to EAE (*p* < 0.001). The rest of the brain treatments show a significant decrease with EAE+TMS without differences with EAE (Figure 5).

**Figure 6:**
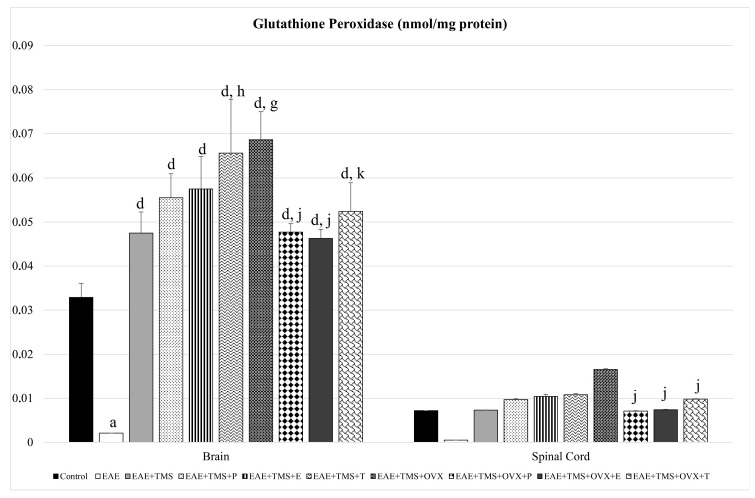
Mean ± standard deviation in glutathione peroxidase (nmol/mg protein) in EAE rats treated with TMS plus hormonal therapy in the following groups: control; EAE; EAE+TMS; EAE+TMS+P; EAE+TMS+E; EAE+TMS+T; EAE+TMS+OVX; EAE+TMS+OVX+P; EAE+TMS+OVX+E; and EAE+TMS+OVX+T in brain and spinal cord. ^a^ *p* < 0.001 vs. control; ^d^ *p* < 0.001 vs. EAE; ^g^ *p* < 0.001 vs. EAE+TMS; ^h^ *p* < 0.01 vs. EAE+TMS; ^j^ *p* < 0.001 vs. EAE+TMS+OVX; ^k^ *p* < 0.01 vs. EAE+TMS+OVX. EAE: experimental autoimmune encephalomyelitis; TMS: transcranial magnetic stimulation; OVX: ovariectomized rats; P: progesterone; E: estrogen; T: testosterone.

As for GPx, only in the brain is there a significant reduction of this enzyme, with EAE, with respect to the control (*p* < 0.001). All brain treatments (TMS, OVX, and hormones) increase GPx with respect to EAE (*p* < 0.001). EAE+TMS+T and EAE+TMS+OVX show higher values of this enzyme than the EAE+TMS group. The application of the three hormonal treatments in the brain, EAE+TMS+OVX+P; EAE+TMS+OVX+E and EAE+TMS+OVX+T, significantly decreases GPX levels in ovariectomized rats treated with TMS with respect to EAE+TMS+OVX. Although in the spinal cord, EAE has not led to significant changes in GPx compared to the control, it has been observed that in the EAE group, there is a tendency towards a 93% decrease in GPx values. For their part, all treatments show a tendency to increase these values by 93–98%, compared to the EAE group. In the EAE+TMS+OVX+P, EAE+TMS+OVX+E, and EAE+TMS+OVX+T groups, GPx is significantly decreased compared to the EAE+TMS+OVX group (Figure 6).

### 3.3. Oxidative Stress Biomarkers

In the spinal cord and brain, EAE causes a significant increase in LPO values compared to the control group (*p* < 0.001). With the different treatments (TMS, OVX, and hormones) in both organs, LPO values are reduced. In the brain and spinal cord, TMS with hormonal treatments (EAE+TMS+P; EAE+TMS+E and EAE+TMS+T) together with TMS+OVX and hormonal treatments (EAE+TMS+OVX+P; EAE+TMS+OVX+E and EAE+TMS+OVX+T) show significantly higher values for LPO than EAE+TMS. Estrogens, progesterone, and testosterone combined with TMS and OVX (EAE+TMS+OVX+E; EAE+TMS+OVX+P and EAE+TMS+OVX+T), have higher levels of LPO in the brain than EAE+TMS+OVX. High LPO values are also observed in EAE+TMS+OVX+E and EAE+TMS+OVX+T in the spinal cord, both compared to EAE+TMS+OVX without hormones (Table 1).

As for CP, both in the brain and spinal cord, they appear significantly increased in EAE with respect to control (*p* < 0.001). In both organs, the different treatments significantly decrease CP values with respect to EAE (*p* < 0.001). In the brain, CP levels in the EAE+TMS+P, EAE+TMS+E, EAE+TMS+T, EAE+TMS+OVX+P, EAE+TMS+OVX+E, and EAE+TMS+OVX+T groups are significantly higher than in EAE+TMS. Also, in the brain, the EAE+TMS+OVX+E and EAE+TMS+OVX+T groups show higher CP values than EAE+TMS+OVX (Table 1).

## 4. Discussion

The aim of this manuscript has been to differentiate the role of sex hormones in two different contexts, one as adjuvants to a treatment and the other as replacement therapy, when the natural hormonal effects are lacking. The results obtained in this research confirm the antioxidant power of TMS on the brain and spinal cord in EAE, through activation of the glutathione redox system. Furthermore, sex hormones, especially estrogen and progesterone, used as hormone replacement therapy in ovariectomized EAE rats, appear to enhance the effects of TMS. Finally, TMS and hormones, in both intact and castrated rats, seem to produce an improvement in the clinical condition of EAE.

Until now, the role of sex hormones in MS has been highly discussed, with contradictory results regarding the modification of the symptoms of MS with menopause, the use of sex hormones as replacement therapy in menopause [18,19], or even the role of sex hormones as a supplementary therapy [39,40].

In this research, in two of the four groups of rats (EAE+TMS+OVX and EAE+TMS+OVX+E), ovariectomy seems to have an effect on EAE induction, since, at 14 days, the clinical score, although above two, is below the rest of the groups. We have not found any literature in this regard. Yet, the effect of menopause on the course of MS (shown by the decrease in the EDSS) has been evaluated with contradictory results. Thus, while Baroncini et al. (2019) observed that menopause increased EDSS, Ladeira et al. (2018) obtained that EDSS progression persists at a similar rate compared to the premenopausal period [41,42]. In the study by Bove et al. (2016), menopause represented a turning point in EDSS changes, increasing EDSS each year after menopause compared to the non-menopausal period [43]. However, no data were found regarding what was obtained in our study.

At 35 days, there is a reduction in clinical score with all treatments except in ovariectomized rats, in which only progesterone replacement therapy is as effective as TMS in uncastrated rats. However, estrogens in ovariectomized rats also reduce clinical symptoms. Milosevic et al. (2020, 2023) demonstrated that with the peak of EAE, hormonal changes occurred both at the serum level and in the ovarian tissue itself [23,44]. In this sense, Milosevic et al. (2020) [44] have shown, in female rats, a significant decrease in LH levels and an increase in progesterone levels at the onset and at the peak of the disease, and unchanged estradiol levels. Furthermore, progesterone levels in steroid samples extracted from ovarian tissue increased significantly at the peak of EAE compared to control levels. In contrast, estradiol levels decreased significantly at peak EAE compared to control values. These hormonal changes could be aggravated in the ovariectomized rats in our study, although no changes were observed in the symptoms of MS due to the use of hormone replacement therapy (HRT). HRT has shown inconclusive results in the clinical symptoms of MS. Holmqvist et al. (2006) conducted a survey on MS symptomatology in menopausal women who used HRT (n = 29), versus those who did not use it, finding that 12 (41%) experienced a worsening of MS symptoms, 16 (55%) reported no change and 1 (3%) improved their symptoms [45]. Smith and Studd (1992) reported an increase in symptom severity with HRT, while Karageorgiou et al. (2020), in a systematic review with menopausal patients with MS, reported an inconclusive association between age at menopause, use of HRT, and disease severity [46,47]. In our study, the addition of progesterone in ovariectomized rats seems to exert an identical response to TMS treatment in uncastrated rats. Taylor et al. (2023), demonstrated in a study on possible fluctuations in the menstrual cycle in MS symptoms, that in patients with an endogenous cycle (hormonal intrauterine device and non-users of oral contraceptives), fatigue was lower (*p* < 0.05) in the perimenstrual period than in the luteal period, which is characterized by the secretion of progesterone [48]. However, it was observed that the risk of developing neurodegenerative disorders was lower in women exposed to formulations containing estrogens with natural progesterone compared to those exposed to products combining estrogens and synthetic progesterone (i.e., medroxyprogesterone acetate). In this regard, medroxyprogesterone acetate (MPA), a synthetic derivative of 17α-hydroxyprogesterone, did not provide neuroprotective effects, and animals with EAE showed as severe a disease as in the control group.

On the other hand, in this work, the estrogen supplement in ovariectomized rats does not reach the effectiveness of TMS alone, but it does reduce the clinical score produced by the EAE.

Regarding the oxidative stress generated by EAE, in our manuscript, treatment with TMS reduces the levels of lipoperoxides and carbonylated proteins (LPO and CP), both in ovariectomized rats and in sexually active rats. However, when TMS is accompanied by hormonal therapy, it appears to reduce its effects compared to simple therapy, both in ovariectomized rats and in sexually active rats. However, divergences in response to hormone treatment have been found, in turn, for antioxidant systems. In this sense, we observed that there is no significant response from any of the treatments in the brain for tG and GSSG or for GSH and GPx in the spinal cord. In all other cases, the antioxidant system of glutathione improves its antioxidant capacity with TMS, with or without hormones, and even HRT, especially estrogen and progesterone, appear to be more effective than TMS alone in castrated rats. This is observed in the spinal cord, where HRT with estrogen and progesterone in castrated rats increases the GSH/GSSG ratio due to a significant reduction in GSSG. In uncastrated rats, TMS alone or in combination with estrogen also improves antioxidant capacity with an increase in the GSH/GSSG ratio, but in this case, this is due to significant increases in GSH levels while GSSG levels remain similar to the control.

The role of sex hormones has been described in MS and EAE without conclusive results. Cerebrovascular inflammation as a result of the autoimmune response of MS has been described to lead to disruption of the blood–brain barrier (BBB), facilitating transendothelial leukocyte migration and increasing axon demyelination and MS symptoms. Transendothelial leukocyte migration has been identified as a part responsible for the inflammation and subsequent oxidative stress of MS [49]. In addition, estrogen depletion is known to increase BBB permeability in female rats and mice, and it has even been suggested that estrogens are important in maintaining BBB integrity. In fact, in response to elevated hydrostatic pressure, the BBB of ovariectomized female rats shows a 500% increase in permeability compared to that of rats with intact ovaries. With the administration of estrogen, the differences between the group treated with estrogen and that of rats with intact ovaries disappear [50]. Specifically, following treatment with 2 µg/kg estrogen per day, endothelial cells extracted from the brain of female mice show increased expression of the tight junction protein, claudin-5, and increased transendothelial electrical resistance, suggesting that estrogen is important for maintaining BBB integrity [51,52].

Furthermore, in the EAE model, estrogens significantly decrease inflammation in the CNS through the estrogen receptor-α (Erα), while their action on Erβ leads to the repair of myelin and axons. The administration of estradiol in phase 2 trials resulted in a decrease in the clinical activity of the disease, despite the detection of inflammatory factors in intracranial magnetic resonance imaging [53]. It has been shown that estrogens can improve some diseases (e.g., sepsis, mood disorders, cerebral ischemia, some liver diseases, Parkinson’s disease, amyotrophic lateral sclerosis, inflammatory bowel disease, spinal cord injury, multiple sclerosis, myocardial ischemia/reperfusion injury, osteoarthritis, and renal fibrosis) by inhibiting nucleotide-binding oligomerization domain inflammasome-like receptor protein 3 (NLRP3) [54]. In one study, administration of estrogen along with immunization against EAE led to an attenuation of the clinical severity of EAE and protected mice against the disease. Administration of both 2000 pg/mL estradiol and 10,000 pg/mL estradiol to mice with EAE resulted in a significant decrease in the percentages of Th1 and Th17 cells. Additionally, the percentages of Th2 and Treg cells increased in the CNS and peripheral lymphoid organs [55].

In turn, testosterone has a protective effect on cultured neurons against glutamate-induced toxicity and oxidative stress. Likewise, it stimulates the formation and regeneration of myelin mediated through the neural androgen receptor. However, in vivo, its effects have been limited [53]. In female mice, preventive androgen administration beneficially controlled T-cell-mediated spleen secretion of the proinflammatory and anti-inflammatory cytokines IFN-γ and IL-10, respectively [56,57]. In addition, androgens in vitro prevented contact between female EAE T cells and astrocytes responsible for the production of proinflammatory molecules [58].

Finally, in a mouse model of MS induced by autoimmune encephalomyelitis, progesterone reduced the infiltration of inflammatory cells into the injured spinal cord, prevented demyelination, and attenuated disease severity. In a mouse model of demyelination, the effects of progesterone on microglial cells are reported to involve a switch from the M1 (pro-inflammatory) to the M2 (anti-inflammatory) phenotype and suppression of the NLRP3 inflammasome [59,60]. Furthermore, in a rat model of spinal cord injury, progesterone treatment after injury increased OPC proliferation and upregulated the mRNA levels of the transcription factors Olig2 and Nkx2.2, which are required for oligodendrocyte differentiation. In addition, Olig1 is involved in myelin repair. Progesterone also increased mRNA and protein levels of myelin basic protein and proteolipid protein. These effects resulted in an increase in mature oligodendrocytes and remyelination [60,61].

However, despite the favorable results obtained in the literature for the three hormonal therapies in the treatment of inflammation and oxidative stress of MS, neither estrogens, nor progesterone, nor testosterone has been shown to be more effective than TMS, nor in normal or castrated rats, in the recovery of oxidative stress in this work. Only as replacement therapy, estrogens and progesterone seem to collaborate with TMS in castrated rats in recruiting antioxidant defenses. This could be due to the nuclear translocation of the Nrf2 factor, causing the expression of antioxidant genes that restore the antioxidant balance and block neuroinflammation [7] or due to the anti-inflammatory power of both hormones through the suppression of the NLPR3 inflammasome [54,59,60]. Such replacement therapy, estrogen and progesterone, has also been very helpful in improving clinical signs of the disease along with TMS, which seems to enhance the effects of female hormones versus male hormones (testosterone) against EAE. This is particularly interesting, as Milosevic et al. (2023) [23] observed a decrease in ovarian testosterone versus a slight increase in serum testosterone in rats with EAE. This increase in serum testosterone could be due, according to the same authors, to an increase in this hormone is attributed to its lack of conversion to estrogen in non-classical steroidogenic organs, such as white adipose tissue (WAT), since EAE causes a drastic reduction in WAT in mice. Therefore, testosterone supplementation may not be relevant in EAE, as it could increase its concentration naturally.

It is worth noting that this study may have several limitations, including: (1) the absence of certain experimental groups, such as the EAE+OVX group without TMS or similar groups receiving only hormones (without TMS), which would provide a clearer understanding of how OVX and hormones alone affect the pathology in the EAE model; (2) the small number of animals per group could affect the statistical power of the study and increase the risk of overinterpretation of the data; and (3) the lack of verification of the molecular pathways through which TMS acts in conjunction with hormone therapy.

Therefore, despite the limitation of this study due to the small number of animals included in each group and the lack of verification of the molecular pathways through which TMS acts together with hormonal therapy, it may be indicated that (1) HRT, especially estrogen and progesterone, in ovariectomized rats is necessary for TMS to be functionally active in improving the clinical signs of the disease; (2) however, the addition of hormones has no more beneficial effects on the clinical score than TMS alone. (3) In the treatment of oxidative stress in EAE, the addition of hormones or HRT does not make TMS more effective. (4) Progesterone and estrogen, as HRT in ovariectomized rats, enhance the role of TMS in the activation of antioxidant defenses, without hormonal supplementation having a relevant role for TMS that works on its own. (5) The role of hormonal supplements does not make TMS more active against EAE, although HRT, especially estrogen and progesterone, is useful to enhance the role of TMS in ovariectomized rats, as far as to the activation of antioxidant defenses and the improvement of the clinical signs of the disease.

## 5. Conclusions

In conclusion, TMS used in menopausal women with multiple sclerosis could be more effective if HRT with estrogens and/or progesterone were used. However, hormonal supplements are not necessary in premenopausal women for TMS to be fully effective.

## Figures and Tables

**Table 1:** Mean ± standard deviation in oxidative stress biomarkers, lipid peroxidation products (LPO; nmol/mg protein), and carbonylated proteins (CP; nmol/g protein) in EAE rats treated with TMS plus hormonal therapy in the following groups: control; EAE; EAE+TMS; EAE+TMS+P; EAE+TMS+E; EAE+TMS+T; EAE+TMS+OVX; EAE+TMS+OVX+P; EAE+TMS+OVX+E; and EAE+TMS+OVX+T in brain and spinal cord. ^a^ *p* < 0.001 vs. control; ^d^ *p* < 0.001 vs. EAE; ^g^ *p* < 0.001 vs EAE+TMS; ^j^ *p* < 0.001 vs. EAE+TMS+OVX; ^l^ *p* < 0.05 vs. EAE+TMS+OVX. EAE: experimental autoimmune encephalomyelitis; TMS: transcranial magnetic stimulation; OVX: ovariectomized rats; P: progesterone; E: estrogen; T: testosterone.

Oxidative Stress Biomarkers		
Brain		
	LPO (nmol/mg protein)	CP (nmol/g protein)
Control	0.8308 ± 0.0005	0.0100 ± 0.0002
EAE	1.8276 ± 0.0029 ^a^	0.0414 ± 0.0001 ^a^
EAE+TMS	0.7224 ± 0.0006 ^d^	0.0088 ± 0.0002 ^d^
EAE+TMS+P	1.1434 ± 0.0011 ^d,g^	0.0110 ± 0.0002 ^d^
EAE+TMS+E	1.1360 ± 0.0046 ^d,g^	0.0207 ± 0.0003 ^d,g^
EAE+TMS+T	1.2668 ± 0.0117 ^d,g^	0.0301 ± 0.0001 ^d,g^
EAE+TMS+OVX	0.7938 ± 0.0042 ^d,g^	0.0111 ± 0.0002 ^d^
EAE+TMS+OVX+P	1.0346 ± 0.0081 ^d,g,j^	0.0142 ± 0.0004 ^d,g^
EAE+TMS+OVX+E	1.1222 ± 0.0097 ^d,g,j^	0.0163 ± 0.0001 ^d,g,l^
EAE+TMS+OVX+T	1.3182 ± 0.0068 ^d,g,j^	0.0258 ± 0.0074 ^d,g,j^
Spinal cords		
Control	0.7037 ± 0.0120	0.0138 ± 0.0001
EAE	2.5419 ± 0.0010 ^a^	0.0335 ± 0.0010 ^a^
EAE+TMS	0.5343 ± 0.0016 ^d^	0.0139 ± 0.0000 ^d^
EAE+TMS+P	0.8411 ± 0.0040 ^d,g^	0.0137 ± 0.0001 ^d^
EAE+TMS+E	1.9490 ± 0.0082 ^d,g^	0.0137 ± 0.0001 ^d^
EAE+TMS+T	1.4143 ± 0.0017 ^d,g^	0.0136 ± 0.0001 ^d^
EAE+TMS+OVX	0.6943 ± 0.0054 ^d,g^	0.0138 ± 0.0001 ^d^
EAE+TMS+OVX+P	0.6774 ± 0.0014 ^d,g^	0.0135 ± 0.0001 ^d^
EAE+TMS+OVX+E	0.8270 ± 0.0017 ^d,g,j^	0.0137 ± 0.0001 ^d^
EAE+TMS+OVX+T	1.5142 ± 0.00152 ^d,g,j^	0.0138 ± 0.0001 ^d^

## Data Availability

The original contributions presented in this study are included in the article/supplementary material. Further inquiries can be directed to the corresponding authors.

## Supplementary Materials

The following supporting information can be downloaded at: https://www.mdpi.com/article/10.3390/biom15121714/s1. Table S1. Mean ± Standard Deviation of the change in clinical score in rats at14 days after EAE induction with MOG; at 35 days of illness and increase between the score at 35 days—score at 14 days, in the groups: vehicle (100 mL of complete Freund’s adjuvant without MOG); EAE+Mock (treated in the same way as those in the TMS group but without receiving real stimulation) and EAE+TMS+Sham (sham-operated) in brain and spinal cord. Table S2. Mean ± Standard Deviation in Glutathione Redox System: total glutathione (tG; nmol/mg protein), reduced glutathione (GSH; nmol/mg protein), oxidized glutathione (GSSG; nmol/mg protein), glutathione peroxidase (GPx; nmol/mg pro-tein) and the ratio between GSH/GSSG in EAE rats in the following groups: vehicle (100 mL of complete Freund’s adjuvant without MOG); EAE+Mock (treated in the same way as those in the TMS group but without receiving real stimulation) and EAE+TMS+Sham (sham- operated) in brain and spinal cord. Table S3. Mean ± Standard Deviation in Oxidative Stress Biomarkers: Lipid peroxidation products (LPO; nmol/mg protein) and carbonylated proteins (CP; nmol/g protein) in EAE rats in the following groups: vehicle (100 mL of complete Freund’s adjuvant without MOG); EAE+Mock (treated in the same way as those in the TMS group but without receiving real stimulation) and EAE+TMS+Sham (sham-operated) in brain and spinal cord.

## Abbreviations

The following abbreviations are used in this manuscript:

TMS	Transcranial magnetic stimulation
MS	Multiple sclerosis
CNS	Central nervous system
EAE	Experimental autoimmune encephalomyelitis
MOG	Myelin oligodendrocyte glycoprotein
PBS	Phosphate buffered saline
E	Estrogens
P	Progesterone
T	Testosterone
OVX	Ovariectomy
LPO	Lipid peroxidation products
tG	Total glutathione
GSH	Reduced glutathione
GSSG	Oxidized glutathione
GPx	Glutathione peroxidase
CP	Carbonylated proteins
EDSS	Expanded disability status scale
HRT	Hormone replacement therapy
BBB	Blood–brain barrier
NLRP3	Inflammasome-like receptor protein 3
