# Supplementary material for: The Impact of Sex Hormones on Transcranial Magnetic Stimulation Against the Oxidative Stress in the Pathogenesis of Multiple Sclerosis
Source: Biomolecules. 2025 Dec 10;15(12):1714. doi: 10.3390/biom15121714 (PMC12730606; doi:10.3390/biom15121714)
Supplement: Supplementary file 1 [file biomolecules-15-01714-s001.zip › biomolecules-3972308-supplementary.pdf]

Clinical Score			
	14 days	35 days	35-14 days
Vehicle	0.000±0.000	0.000±0.000	0.000±0.000
EAE+Mock	3.200±0.837	4.000±0.000	0.800±0.837
EAE+TMS+Sham	2.750±0.4330	2.500±0.500	-0.250±0.433

Table S1. Mean ± Standard Deviation of the change in clinical score in rats at14 days after EAE induction with MOG; at 35 days of illness and increase between the score at 35 days – score at 14 days, in the groups: vehicle (100 ml of complete Freund's adjuvant without MOG); EAE+Mock (treated in the same way as those in the TMS group but without receiving real stimulation) and EAE+TMS+Sham (sham-operated) in brain and spinal cord.

EAE: experimental autoimmune encephalomyelitis; TMS: transcranial magnetic stimulation; MOG: myelin oligodendrocyte glycoprotein

Glutathione Redox System			
**Brain**			
	Vehicle	EAE+Mock	EAE+TMS+Sham
tG (nmol/mg protein)	0.010±0.000	0.019±0.010	0.010±0.002
GSH (nmol/mg protein)	0.009±0.000	0.009±0.002	0.008±0.001
GSSG (nmol/mg protein)	0.001±0.000	0.011±0.008	0.002±0.000
GSH/GSSG	7.607±1.150	1.518±1.217	3.992±0.364
GPx (nmol/mg protein)	0.046±0.012	0.002±0.000	0.057±0.003
**Spinal cord**			
	Vehicle	EAE+Mock	EAE+TMS+Sham
tG (nmol/mg protein)	0.010±0.000	0.034±0.004	0.013±0.002
GSH (nmol/mg protein)	0.008±0.000	0.009±0.000	0.010±0.001
GSSG (nmol/mg protein)	0.001±0.000	0.025±0.004	0.003±0.000
GSH/GSSG	5.631±0.211	0.383±0.051	3.625±0.138
GPx (nmol/mg protein)	0.007±0.000	0.001±0.000	0.011±0.000

Table S2. Mean ± Standard Deviation in Glutathione Redox System: total glutathione (tG; nmol/mg protein), reduced glutathione (GSH; nmol/mg protein), oxidized glutathione (GSSG; nmol/mg protein), glutathione peroxidase (GPx; nmol/mg pro-tein) and the ratio between GSH/ GSSG in EAE rats in the following groups: vehicle (100 ml of complete Freund's adjuvant without MOG); EAE+Mock (treated in the same way as those in the TMS group but without receiving real stimulation) and EAE+TMS +Sham (sham-operated) in brain and spinal cord.

EAE: experimental autoimmune encephalomyelitis; TMS: transcranial magnetic stimulation; MOG: myelin oligodendrocyte glycoprotein

Oxidative Stress Biomarkers			
**Brain**			
	Vehicle	EAE+Mock	EAE+TMS+Sham
LPO (nmol/mg protein)	0.820±0.006	1.822±0.008	0.826±0.007
CP (nmol/g protein)	0.010±0.000	0.082±0.000	0.011±0.000
**Spinal cord**			
LPO (nmol/mg protein)	0.691±0.017	2.540±0.004	0.694±0.005
CP (nmol/g protein)	0.004±0.000	0.132±0.000	0.004±0.000

Table S3. Mean ± Standard Deviation in Oxidative Stress Biomarkers: Lipid peroxidation products (LPO; nmol/mg protein) and carbonylated proteins (CP; nmol/g protein) in EAE rats in the following groups: vehicle (100 ml of complete Freund's adjuvant without MOG); EAE+Mock (treated in the same way as those in the TMS group but without receiving real stimulation) and EAE+TMS+Sham (sham-operated) in brain and spinal cord.

EAE: experimental autoimmune encephalomyelitis; TMS: transcranial magnetic stimulation; MOG: myelin oligodendrocyte glycoprotein
